# Efficacy and safety analysis of China’s first 10% IVIg (RonsenGlob) therapy in treating adult ITP

**DOI:** 10.1007/s00277-025-06391-1

**Published:** 2025-05-10

**Authors:** Lijun Fang, Ting Sun, Hu Zhou, Guitao Jie, Jiaping Fu, Enqin Yang, Zeping Zhou, Ligen Liu, Jingyu Zhang, Shenxian Qian, Yun Chen, Ling Liu, Jian Gu, Fanliang Kong, Ruibin Huang, Yunfei Chen, Lei Zhang

**Affiliations:** 1 State Key Laboratory of Experimental Hematology, National Clinical Research Center for Blood Diseases, Haihe Laboratory of Cell Ecosystem, Tianjin & CAMS Key Laboratory of Gene Therapy for Blood Diseases, Institute of Hematology & Blood Diseases Hospital, Chinese Academy of Medical Sciences & Peking Union Medical College, Tianjin, 300020 China; 2 Tianjin Institutes of Health Science, Tianjin, 301600 China; 3 School of Population Medicine and Public Health, Chinese Academy of Medical Sciences and Peking Union Medical College, Beijing, 100730 China; 4 The Affiliated Cancer Hospital of Zhengzhou University & Henan Cancer Hospital, Zhengzhou, 450000 China; 5 Linyi Central Hospital, Linyi, 276000 China; 6 Shaoxing People’s Hospital, Shaoxing, 312000 China; 7 People’s Hospital of Rizhao, Rizhao, 276800 China; 8 The Second Affiliated Hospital of Kunming Medical University, Kunming, 650000 China; 9 Tong Ren Hospital, Shanghai Jiao Tong University School of Medicine, Shanghai, 200000 China; 10 The Second Hospital of Hebei Medical University, Shijiazhuang, 050000 China; 11 Affiliated Hangzhou Frist Peoples Hospital, School of Medicine, Westlake University, Hangzhou, 310000 China; 12 Central Hospital Affiliated to Shandong First Medical University, Jinan, 250000 China; 13 The Second Affiliated Hospital of Guangzhou Medical University, Guangzhou, 510000 China; 14 Northern Jiangsu People’s Hospital, Yangzhou, 225000 China; 15 The Second People’s Hospital of Hefei, Hefei, 230000 China; 16 The First Affiliated Hospital of Nanchang University, Nanchang, 330000 China

**Keywords:** Immune thrombocytopenic purpura, Intravenous immunoglobulin (IVIg), Clinical trial, Efficacy and safety

## Abstract

A prospective, single-arm, open-label Phase III clinical trial was conducted across multiple centers in China from April 27, 2020, to June 15, 2021, to assess the efficacy and safety of 10% intravenous immunoglobulin (IVIg) in treating adult immune thrombocytopenic purpura (ITP). Within 7 days of treatment initiation, the 10% IVIg group exhibited an overall response rate of 87.0%, with 32 patients (46.4%) achieving complete response and 28 patients (40.6%) demonstrating partial response, comparable to the 5% IVIg group. Notably, the median time to achieve a platelet count (PLT) of 50 × 10^9^/L was significantly shorter for the 10% IVIg group at 2 days (IQR: 2–3) versus 3 days (IQR: 3–5) for the 5% IVIg group. Additionally, the 10% IVIg group reached a PLT of 100 × 10^9^/L in 3 days (IQR: 3–4), compared to 5 days (IQR: 4–6) for the 5% IVIg group. Post-treatment bleeding scores significantly decreased, and no significant adverse reactions were reported. This inaugural study highlights the efficacy and safety of 10% IVIg in the urgent management of adult ITP, positioning it as a rapid therapeutic option.

**Supplementary Information:**

The online version contains supplementary material available at 10.1007/s00277-025-06391-1.

## Introduction

Primary Immune Thrombocytopenia (ITP) is an acquired autoimmune hemorrhagic disorder characterized by a broad spectrum of clinical manifestations, ranging from asymptomatic thrombocytopenia to severe bleeding, predominantly mucocutaneous hemorrhage. In severe cases, visceral or intracranial bleeding may occur, with the risk of bleeding increasing with age. The pathogenesis of ITP primarily involves immune intolerance to self-antigens, resulting in enhanced immune-mediated destruction of platelets and reduced platelet production by megakaryocytes [1, 2].

Intravenous Immunoglobulin (IVIg) is a pivotal therapeutic agent for ITP [3], primarily by blocking Fc receptors on mononuclear macrophages, thereby inhibiting antibody-dependent cellular cytotoxicity and reducing platelet destruction [4]. Compared to corticosteroids, IVIg offers advantages such as a rapid onset of action, minimal side effects, and shorter treatment duration. For certain elderly patients with impaired cardiac function or those with a high body weight, and especially in emergency treatments for severe ITP complicated by critical organ bleeding (such as intracranial or gastrointestinal hemorrhage) or pregnancy-associated ITP with fetal risk, excessive fluid infusion may increase potential treatment-related risks [5, 6, 7, 8]. Therefore, higher-concentration IVIg formulations can help reduce these potential risks, ultimately delivering greater clinical benefits to patients with diverse needs.

High-concentration IVIg (pH4, 10%) treatments for ITP have demonstrated favorable efficacy and safety internationally [9], partially ameliorating these potential risks. Nonetheless, clinical trials evaluating their efficacy and safety in China are lacking. Therefore, this study initiated a prospective, multicenter, open clinical trial to evaluate the clinical efficacy and safety of China’s first independently developed 10% IVIg (RonsenGlob) for ITP.

## Materials and methods

### Study design

**Fig. 1:**
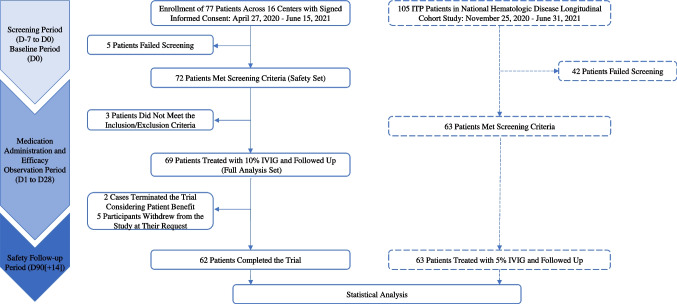
Clinical trial flowchart. Flow diagram illustrating the enrollment, screening, eligibility, treatment, follow-up, and final inclusion of patients in the 10% IVIg prospective clinical trial and the concurrently enrolled 5% IVIg cohort from the national hematologic disease longitudinal study, selected based on identical inclusion and exclusion criteria

To evaluate the efficacy and safety of IVIg (pH4, 10%) (chromatography) in patients with ITP, we conducted a multicenter, open-label clinical study titled “An Open-Label, Multicenter Clinical Study of the Efficacy and Safety of Intravenous Human Immunoglobulin (pH4, 10%) (Chromatography) in Patients with Primary Immune Thrombocytopenia,” registered under ChiCTR2300069388. This single-arm, Phase III clinical trial spanned from April 27, 2020, to June 15, 2021, across 16 clinical centers nationwide, focusing on adult ITP treatment with 10% IVIg. Additionally, to assess the efficacy and safety differences between the 10% IVIg (1 g/kg/d for 2 days) and 5% IVIg (0.4 g/kg/d for 5 days) treatment regimens, 63 ITP patients treated with the 5% IVIg regimen from a simultaneous prospective national hematology disease longitudinal cohort study (ClinicalTrials.gov ID: NCT04645199) were included based on the 10% IVIg trial’s inclusion and exclusion criteria (Details of the two studies are in the Supplement Results). The clinical characteristics, efficacy, and safety of both patient groups were compared (The study flow chart is shown in Fig. 1).

### Study population

**Inclusion criteria:** Patients aged 18–65 years (inclusive), with a disease duration of more than 3 months, PLT < 30 × 10^9^/L, and patients who have been using a fixed dose of steroids for two weeks or more prior to the baseline period, or patients with ITP who have not used steroids for at least two weeks. **Exclusion criteria**: 1. Patients with refractory ITP (according to the"Consensus of Chinese Experts on Diagnosis and Treatment of Adult Primary Immune Thrombocytopenia"(Version 2016)); 2. Previously failed immunoglobulin therapy or known/suspected allergy to human immunoglobulin or corticosteroids; 3. Patients who received rituximab treatment within 3 months prior to enrollment; 4. Patients who received treatment with immunosuppressants, TPO-raising drugs, or other medications known to increase platelet counts within 4 weeks before the baseline; 5. Patients who had transfusions or used blood products, including immunoglobulins, within 30 days before the baseline; 6. Patients with severe liver or kidney diseases or abnormal liver and kidney function tests; 7. Post-splenectomy patients, etc. (Detailed inclusion and exclusion criteria are available as Supplementary Results Part 1).

### Sample size

In the 10% IVIg trial, a total of 77 subjects signed 82 informed consent forms (ICFs). Among them, 10 subjects failed the initial screening. Five of these subjects, after failing the first screening, re-signed the ICF and were re-screened, meeting the protocol requirements. A total of 72 subjects passed the screening and entered the *Safety Set (SS)*. According to the decision of the data review committee, 3 subjects who did not meet the protocol criteria were excluded from the 72 subjects, leaving 69 subjects who met the protocol and entered the *Full Analysis Set (FAS)*. All 69 subjects received at least one dose of the study drug and had at least one post-treatment platelet count result. All enrolled subjects completed the study treatment. Following the inclusion and exclusion criteria and treatment protocols of the 10% IVIg clinical trial had platelet monitoring data up to 28 days post-treatment, only 63 eligible patients from the national hematology disease longitudinal cohort study during the same period were included in the 5% IVIg group.

*Safety Set (SS): Comprises all subjects who received at least one dose of the study drug, utilized for the analysis of treatment safety.
*Full Analysis Set (FAS): Includes all enrolled subjects who received at least one dose of the study drug and had at least one post-treatment platelet count, used for the analysis of clinical baseline characteristics and efficacy.

### Drug administration and follow-up

5% IVIg group: IVIg (2.5 g per bottle (5%, 50 ml)), intravenous drip, 0.4 g/kg/d, infused for 5 consecutive days.

10% IVIg group: IVIg (5.0 g per bottle (10%, 50 ml)), intravenous drip, 1.0 g/kg/d, infused for 2 consecutive days. The main efficacy observation period is the first 7 days (up to the visit on the 8 th day), with follow-up visits for efficacy and safety evaluations at the study center on days 8, 14, 21, and 28. On day 90 after treatment, participants return to the study center for tests of infectious markers, pregnancy tests, collection of adverse event (AE) information, and concomitant medication data.

### Efficacy evaluation

#### Primary endpoint

Overall response (OR) rate of subjects within 7 days after starting treatment. OR was defined as the percentage of patients with complete response (CR) or partial response (PR) after treatment^*^.

#### Secondary efficacy endpoints

1. Percentage of subjects with PLT reaching 50 × 10^9^/L and 100 × 10^9^/L or above within 7 days after starting treatment, the time (in days) to reach these levels, and the duration of this response.
2. OR rate (%) of subjects on days 14, 21, and 28 after starting treatment.
3. Bleeding scores before treatment and 7 days after treatment, scored according to the bleeding score system in the"Consensus of Chinese Experts on Diagnosis and Treatment of Adult Primary Immune Thrombocytopenia"(Version 2016).

(Detailed efficacy evaluation criteria are included in Supplementary Result 1.)

*Complete response (CR): post-treatment PLT ≥ 100 × 10^9^/L. Partial response (PR): post-treatment PLT ≥ 30 × 10^9^/L and at least double the baseline PLT but less than 100 × 10^9^/L. Non-response (NR): post-treatment PLT < 30 × 10^9^/L or PLT increase less than twice the baseline value. Overall response (OR): the total proportion of patients achieving either a CR or PR after treatment.

#### Statistical methods

The FAS was utilized for the analysis of clinical baseline characteristics and efficacy, while the SS was used for safety analysis. Statistical analysis and graphing were conducted using SPSS 22.0 software and GraphPad Prism 8.3.0. Comparisons between groups were made using the T-test, Chi-squared test, Wilcoxon-Mann–Whitney test, and Fisher’s Exact Test. The rank-sum test was used to calculate the median days and duration days for platelet counts first reaching 50 × 10^9^/L, and 100 × 10^9^/L, to compare differences between groups. Clinical baseline information of the patients, baseline levels of platelets and bleeding scores, previous medication use, previous treatments and all the statistical results of efficacy evaluation metrics were expressed as mean ± standard deviation ($\overline{\chi }$ ± SD), medians (interquartile ranges, IQR), and percentages. All tests were two-sided, with *p* < 0.05 considered statistically significant. *: *p* < 0.05.

## Results

### Basline clinical characteristics of patients

The baseline clinical characteristics of 69 patients in the 10% IVIg group’s FAS were statistically analyzed, including age, gender, duration of disease history, baseline platelet levels, bleeding scores, and prior medication history. Similarly, the clinical information for patients in the 5% IVIg group was also analyzed. The results revealed no statistical differences between the two groups in terms of age, gender, duration of disease history, baseline PLT levels, and bleeding scores. However, there were statistical differences in certain aspects of prior medication use, as detailed in Table 1.

**Table 1:** Patient Demographics and Baseline Characteristics ($\overline{\chi }$ ± SD)

Characteristics	5%IVIg (*N* = 63)	10%IVIg (*N *= 69)	*p*
Age (years)	41.1 ± 12.7	42.5 ± 12.9	0.511^a^
Sex, *n* (%)			
Male	23 (36.5)	22 (31.9)	0.707^b^
Female	40 (63.5)	47 (68.1)	
Duration of thrombocytopenia (months)	54.1 ± 90.9	42.5 ± 58.0	0.501^a^
Baseline platelet count (× 10^9^/L)	13 ± 8	11 ± 8	0.341^a^
< 10 × 10^9^/L, *n* (%)	24 (38.1)	33 (47.8)	
≥ 10 × 10^9^/L, *n* (%)	39 (61.9)	36 (52.2)	
Bleeding score, *n* (%)			
0	32 (50.8)	26 (37.7)	0.131^c^
1	23 (36.5)	31 (44.9)	
2	7 (11.1)	9 (13.0)	
3	1 (1.6)	3 (4.3)	
≥ 4	0 (0)	0 (0)	
Previous therapies,			
Glucocorticoid	36	23	0.010^b^
IVIg	29	0	< 0.001^d^
rhTPO/TPO receptor agonists	9	4	0.180^b^
Cyclosporine	3	1	0.348^d^
Danazol	7	5	0.640^b^
Rituximab	2	0	0.226^d^
Vincristine	2	0	0.226^d^

*N* number of patients, *rhTPO* recombinant human thrombopoietin

a: T test

b: Chi-squared test

c: Wilcoxon-Mann–Whitney test

d: Fisher’s Exact Test

### Efficacy evaluation

#### OR evaluation of patients

We calculated the proportion of OR within 7 days of starting treatment, defined as the proportion of patients achieving PR or CR during this period. The OR proportion in the 10% IVIg group was 87.0%, with 32 patients (46.4%) achieving CR and 28 patients (40.6%) reaching PR within 7 days. In the 5% IVIg group, the OR proportion was 82.5%; among them, 32 patients (50.8%) achieved CR, and 20 patients (31.7%) reached PR. No statistical differences were found between the two groups in OR proportion and response evaluation results, as shown in Table 2 and Fig. 3.

**Table 2:** Efficacy Evaluation

	5%IVIg (*N* = 63)	10%IVIg (*N* = 69)	*p*
Primary outcome			
Total response rate within 7 days after initiation of treatment. n(%)^[a]^	52 (82.5)	60 (87.0)	0.643
Secondary outcome			
Proportion of patients achieving a platelet count of 50 × 10^9^/L or higher within 7 days following treatment initiation^[a]^	47 (74.6)	55 (79.7)	0.484
Proportion of patients achieving a platelet count of 100 × 10^9^/L or higher within 7 days following treatment initiation ^[a]^	32(50.8)	32 (46.4)	0.612
Time to first attainment of a platelet count of 30 × 10^9^/L and surpassing double the baseline level within 7 days after initiating treatment. Median duration (Interquartile Range, IQR)^[b]^	3(3,5)	1(1,2)	< 0.001
Time to first attainment of a platelet count of 50 × 10^9^/L within 7 days following treatment initiation. Median duration (Interquartile Range, IQR) ^[b]^	3(3,5)	2(2,3)	< 0.001
Time to first attainment of a platelet count of 100 × 10^9^/L within 7 days of starting treatment. Median time (Interquartile Range, IQR)^[b]^	5(4,6)	3(3,4)	< 0.001
The duration of a first 30 × 10^9^/L platelet count that increased to more than twice the baseline level within 7 days after the initiation of treatment. Median days (IQR)^[b]^	19(12,26)	13(11,19)	0.044
The duration of a first 50 × 10^9^/L platelet count within 7 days after the initiation of treatment. Median days (IQR)^[b]^	17(10,24)	13(6,19)	0.072
The duration of a first 100 × 10^9^/L platelet count within 7 days after the initiation of treatment. Median days (IQR)^[b]^	10(9,24)	11(10,17)	0.437
Total response rate on the 14 th, 21 st, and 28 th day after medication (%)^[a]^			
D14	50.8	30.4	0.027
D21	6.3	15.9	0.144
D28	9.5	4.3	0.405

[a] Using the Chi-squared test

[b] Using the rank sum test

We further analyzed the proportion of patients whose PLT exceeded 50 × 10^9^/L and 100 × 10^9^/L within 7 days to assess platelet recovery after treatment. In the 10% IVIg group, the percentages of patients reaching PLT over 50 × 10^9^/L and 100 × 10^9^/L were 79.7% and 46.4%, respectively; in the 5% IVIg group, these percentages were 74.6% and 50.8%, respectively, with no statistical differences between groups, as shown in Table 2.

Finally, we calculated the OR proportion of subjects on days 14, 21, and 28 after starting medication to evaluate the long-term efficacy. The OR proportions in the 10% IVIg group on days 14, 21, and 28 were 30.4%, 15.9%, and 4.3%, respectively; in the 5% IVIg group, they were 50.8%, 6.3%, and 9.5%, respectively. A statistical difference was observed between groups on day 14, but not on days 21 and 28, as detailed in Table 2 and Fig. 3.

#### Statistics on PLT, onset time, and duration of response

**Fig. 2:**
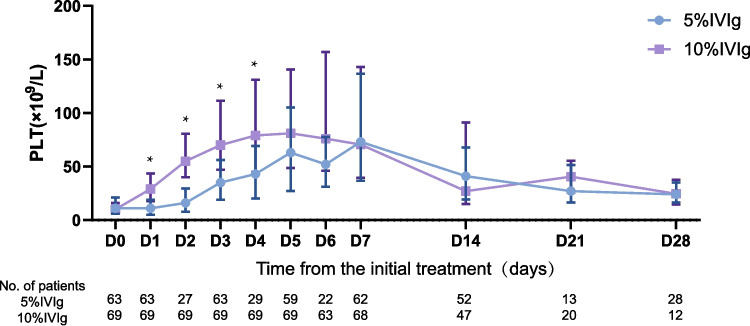
Platelet count changes following IVIg treatment. Median platelet counts (× 10⁹/L) with interquartile range (IQR) are shown from baseline (Day 0) to Day 28 in patients treated with either 5% or 10% IVIg. Asterisks indicate time points with statistically significant differences between groups (*p < 0.05). Number of patients assessed at each time point is shown below the x-axis for each treatment group

**Fig. 3:**
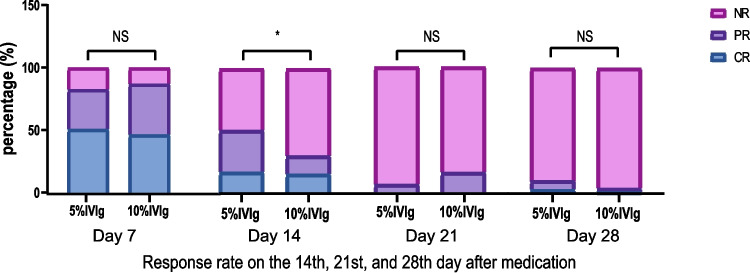
Comparison of response rates on the 14th, 21st, and 28th day after medication. Stacked bar charts showing the proportions of complete response (CR), partial response (PR), and no response (NR) at Days 7, 14, 21, and 28 after starting medication for patients receiving 5% or 10% IVIg. A statistically significant difference was observed on Day 14 (*p < 0.05), with no significant differences (NS) at other time points

We calculated and plotted the median PLT with interquartile range (IQR) before and after treatment (Fig. 2). On days 2–5 of the study (days 1–4 after treatment), platelet counts in the 10% IVIg group were significantly higher than in the 5% IVIg group, indicating a quicker increase in PLT with the 10% IVIg regimen (Fig. 3).

The median time for patients to first reach a PLT of 50 × 10^9^/L within 7 days after treatment was 2 days (IQR: 2, 3) for the 10% IVIg group, lasting for a median of 13 days (IQR: 6, 19). For the 5% IVIg group, the median time was 3 days (IQR: 3, 5), with a response duration of 17 days (IQR: 10, 24). Patients in the 10% IVIg group reached 50 × 10^9^/L significantly earlier than those in the 5% IVIg group (*p* < 0.05), with no significant difference in duration. The median time to first reach a PLT of 100 × 10^9^/L was 3 days (IQR: 3, 4) for the 10% IVIg group, lasting for a median of 11 days (IQR: 10, 17), while for the 5% IVIg group, it was 5 days (IQR: 4, 6), with a response duration of 10 days (IQR: 9, 24). The 10% IVIg group reached 100 × 10^9^/L significantly earlier (*p* < 0.05), with no significant difference in duration, as detailed in Table 2.

These results suggest that 10% IVIg treatment leads to a quicker increase in platelet counts, with an earlier time to response, though the duration of this response does not surpass that of the 5% IVIg group.

#### Bleeding score

The bleeding score, a direct indicator of patient bleeding symptoms, was analyzed (Table S1). In the 10% IVIg group, the number of patients with no bleeding symptoms (bleeding score of 0) increased from 26 (37.7%) before treatment (D0) to 42 (62.7%) after treatment (D7), showing a significant reduction in bleeding scores (*p* < 0.05). Similarly, the bleeding score in the 5% IVIg group also significantly decreased post-treatment (*p* < 0.05). There was no significant difference in bleeding scores between the groups before treatment (D0), but a significant difference was observed after treatment (D7) (*p* < 0.05).

## Safety evaluation

### Treatment-related adverse events (TRAEs) during the treatment period

The most common TRAE in the 10% IVIg group (occurring in ≥ 5% of participants) were: headache (22.2%), decreased white blood cell count (20.8%), decreased neutrophil count (11.1%), increased blood pressure (9.7%), and dizziness (6.9%). In the 5% IVIg group, the rates of drug-related AEs were: headache (11.1%), decreased white blood cells (7.9%), dizziness (6.3%), decreased neutrophil count (4.8%), and fever (4.8%), as detailed in Table 3.

**Table 3:** Treatment-related adverse event

AE, *n* (%)	5%IVIg (*N* = 63) *n* (%)	10%IVIg (*N* = 72) *n* (%)	*p* (χ^2^ test)
Headache	7 (11.1)	16 (22.2)	0.138
White blood cell count decreased	6 (9.5)	15(20.8)	0.116
The neutrophil count decreased	3 (4.8)	8 (11.1)	0.303
Blood pressure increased	2 (3.2)	7(9.7)	0.240
Dizziness	4 (6.3)	5 (6.9)	1.000
Vomiting	2 (3.2)	5 (6.9)	0.551
Fever	3 (4.8)	4 (5.6)	1.000
Rash	2 (3.2)	4 (5.6)	0.802

### Treatment-emergent adverse events (TEAEs) during the treatment period

Among the 72 participants in the 10% IVIg study, the most common TEAE occurring in ≥ 5% of participants were: headache (22.2%), decreased white blood cell count (23.6%), decreased neutrophil count (15.3%), increased blood pressure (11.1%), and hypokalemia (9.7%). In the 5% IVIg group, which included 63 patients, the most common AEs were: headache (15.9%), decreased white blood cell count (11.1%), decreased neutrophil count (7.9%), increased blood pressure (7.9%), and anemia (7.9%), as detailed in Table S4.

No TEAEs led to a reduction in the dose of the study drug, cessation of the study drug, or death in the 10% IVIg group. However, there were 2 cases (2.8%) of serious TEAEs in this group, namely lacunar stroke and acute coronary syndrome, both classified as grade 3 (severe). Upon review, both serious adverse events (SAEs) were determined to be unrelated to the study drug, and both cases recovered without sequelae.

## Discussion

IVIg is a first-line therapy for ITP. Currently, 5% IVIg is commonly used domestically to treat ITP and autoimmune diseases; its clinical use poses challenges, particularly due to potential therapeutic risks associated with large infusion volumes. Internationally, high-concentration IVIg (10%) has been adopted, offering a more efficient and convenient treatment option for ITP patients. To further evaluate the efficacy and safety of 10% IVIg for ITP, we conducted China’s first prospective, open-label, multicenter clinical trial. For comparison, we utilized data from 63 ITP cases treated with the 5% IVIg regimen from a concurrent prospective national hematology cohort study conducted at our center.

The primary efficacy endpoint of this study was the OR proportion within 7 days of treatment initiation. In the 10% IVIg group, the OR proportion was 87.0%, with 87% of patients achieving a PLT of 30 × 10^9^/L or more, which is at least double the baseline value, within 7 days. Furthermore, 79.7% of subjects reached a PLT of 50 × 10^9^/L or higher within the same timeframe. These results align closely with international data for 10% IVIg products: Privigen was 80.7% [10], Flebogamma 10% DIF was 81.3% [11], and Panzyga was 80.6% [12]. Furthermore, we also calculated the OR proportions on days 14, 21, and 28 after medication, which were 30.4%, 15.9%, and 4.3%, respectively. Compared to the 5% IVIg group, there was no significant difference in the OR proportions of the 10% IVIg group at 7, 21, and 28 days. Given that the treatment duration for the 5% IVIg group was longer than that for the 10% IVIg group, the time to peak platelet count was delayed. This might be one of the reasons for the difference in OR rates between the 10% IVIg group and the 5% IVIg group on day 14. Therefore, we conclude that the efficacy of the 10% IVIg treatment regimen is broadly equivalent to that of the 5% IVIg treatment regimen.

In this study, the median time for the 10% IVIg group to first reach a PLT of 50 × 10^9^/L was 2 days (IQR: 2,3), compared to 3 days (IQR: 3,5) in the 5% IVIg group, indicating a statistically significant difference in favor of the 10% regimen. Previous studies have reported similar findings, with a median time of 2 days for adult chronic ITP patients treated with 10% IVIg [12, 13], corroborating our results. Additionally, the median time for the 10% IVIg group to first reach a PLT of 100 × 10^9^/L was 3 days(IQR: 3,4), significantly earlier than in the 5% IVIg group. This suggests that the 10% IVIg regimen can more rapidly increase platelet counts, thereby reducing bleeding risk.

The median duration for maintaining a PLT of 50 × 10^9^/L in the 10% IVIg group was 13 days (IQR: 6,19), consistent with prior studies [12–15], while the duration for maintaining a PLT of 100 × 10^9^/L was 11 days (IQR: 10,17). These durations did not significantly differ from those in the 5% IVIg group, which were 17 days (IQR: 10,24) and 10 days (IQR: 9,24), respectively. Therefore, there is no significant difference in platelet maintenance duration between the two treatment regimens.

The bleeding scores for patients in both groups showed a significant decrease before and after treatment (*p* < 0.05), suggesting that the 10% IVIg treatment regimen can also alleviate patients’bleeding symptoms. However, there was a statistical difference in the bleeding scores of patients after treatment (D7) between the two groups. Since the median bleeding scores for both groups were 0, we consider this statistical difference to not have practical clinical significance.

The common TEAEs in the 10% group included headache (22.2%), decreased white blood cell count (20.8%), decreased neutrophil count (15.3%), blood pressure increased (11.1%), and hypokalemia (9.7%), etc. TRAEs in the 10% group included: headache (22.2%), white blood cell count decreased (20.8%), neutrophil count decreased (11.1%), blood pressure increased (9.7%), and dizziness (6.9%), etc. These are similar to the common adverse reactions reported in previous literature, such as headache, fever, dizziness, nausea, vomiting, increased heart rate, hypertension, etc. [6, 13], with no new or unique adverse reactions identified. Notably, this study observed a relatively high incidence of decreased white blood cell and neutrophil counts, possibly related to anti-neutrophil cytoplasmic antibodies present in IVIg or the immunological clearance mediated by sialic acid/Siglec-9, or potentially associated with the activation of neutrophils by IVIg causing their migration to the vessel wall [16, 17]. Most TEAEs were mild to moderate (CTCAE 5.0 grade 1–3) and generally resolved with symptomatic treatment. Two cases of SAEs were reported in the 10% IVIg group, unrelated to treatment, and resolved without sequelae. The serious adverse reactions observed in the premarketing clinical studys of subjects receiving other IVIG products for ITP were as follows: Privigen (aseptic meningitis syndrome,hemolysis) [10], Gammaplex 5% (vomiting, dehydration and headache) [9], IQYMUNE®(aseptic meningitis, infuenza-like illness, accidental overdose, underdose, vascular encephalopathy with hydrocephalus syndrome) [18]. PANZYGA (aseptic meningitis) [12]. Importantly, this study of RonsenGlob found no serious adverse reactions. Therefore, we believe that the 10% IVIg regimen exhibits a high safety profile.

This study has the following limitation: Firstly, the relatively small sample size of the external control group restricted our ability to perform propensity score matching (PSM) analysis, a statistical method that could have further minimized potential selection bias. Although baseline characteristics between the two groups were generally comparable, the absence of PSM may still influence the reliability and generalizability of our findings. Secondly, due to the limited sample size overall, our study did not achieve an adequate statistical power (post-hoc calculated power of 0.6693), falling short of the commonly recommended threshold of 0.8. This insufficient power might have reduced our capacity to detect certain meaningful differences between groups, thus caution is warranted when interpreting negative results. Future studies with larger sample sizes and more rigorous matching methodologies (such as PSM) are necessary to validate and extend the conclusions drawn from this study.

## Conclusion

This study represents the first application of 10% IVIg in China for treating adult ITP, demonstrating promising efficacy and safety. The treatment facilitates a rapid increase in platelet counts and effectively reduces bleeding symptoms, providing significant clinical benefits for adult ITP patients, particularly those requiring emergency intervention due to bleeding. Clinicians should judiciously arrange medication use based on individual patient requirements and the urgency of clinical situations to enhance treatment efficacy and improve patient experience.

## Supplementary Information

Below is the link to the electronic supplementary material.

ESM 1 (DOCX 32.6 KB)

## Data Availability

No datasets were generated or analysed during the current study.

## Declarations

### Trial registration

The clinical study evaluating 10% IVIg is registered on Chinese Clinical Trial Registry with the registration number ChiCTR2300069388. Additionally, the longitudinal cohort study of 5% IVIg has been registered on ClinicalTrials.gov under the registration number NCT04645199.
