# Supplementary material for: Efficacy and safety analysis of China’s first 10% IVIg (RonsenGlob) therapy in treating adult ITP
Source: Ann Hematol. 2025 May 10;104(5):2643–51. doi: 10.1007/s00277-025-06391-1 (PMC12141155; doi:10.1007/s00277-025-06391-1)
Supplement: Supplementary file 1 — (DOCX 32.6 KB) [file 277_2025_6391_MOESM1_ESM.docx]

**Supplementary Results**

**1. Description for medicine or protocol of treatment in detail of 10% IVIg Group**：

***Inclusion criteria***

1. 18 to 65 years of age (including 18 and 65 years old), male or female; 2. At least 3 months from clinically diagnosis as primary immune thrombocytopenia to signing informed consent; 3. As of screening, patients who have been using fixed doses of glucocorticoids for two weeks or more or who have not used glucocorticoids for at least two weeks; 4. Blood platelet count < 30×10^9^/L; 5. Able to understand the procedures and methods of this study, willing to sign informed consent form and strictly abide by the clinical protocol to complete this study; 6. Agree to take effective contraceptive measures during the trial and within 3 months after the end of the last administration.

***Exclusion criteria***

Refractory ITP (according to "Consensus of Chinese experts on diagnosis and treatment of adult ITP" (Verson 2016); 2. Known or suspected to allergy to or have serious adverse reactions to human immunoglobulin or steroid hormone, or have allergic constitution; 3. Received rituximab within 3 months before enrollment; 4. Received immunosuppressive agent (such as azathioprine, cyclosporine A, cyclophosphamide, vincristine, mycophenolate mofetil, danazol, etc., except glucocorticoids), TPO or other drugs that have the confirmed effect of improving platelet count (such as TPO receptor agonist (receptor agonist Qubopa, Romistine, etc.), IL-11, platelet-ascending capsule, Weixuening, etc.) within 4 weeks before screening; 5. Received blood transfusion or blood products including human immunoglobulin within 30 days before screening; 6. Planning to receive blood transfusion or other blood products during the trial period; 7. Acute bacterial or acute viral infection that still need to be treated with antibiotics after enrollment; 8. Uncontrollable hypertension (SBP > 160mmHg and/or DBP > 100mmHg); 9. Hemoglobin < 6 g/dL; 10. Serious liver and kidney diseases, or abnormal liver and kidney function examination (ALT or AST > 2 times of the upper limit of normal range, or total bilirubin > 1.5 times of the upper limit of normal range, or Cr > 1.5 times of the upper limit of normal range, if any one is satisfied); 11. History of selective IgA deficiency; 12. Positive for hepatitis B surface antigen, hepatitis C antibody, syphilis antibody or HIV antibody; 13. Splenectomy or plan to undergo splenectomy; 14. Previously failed with immunoglobulin therapy; 15. Immune haemolytic anaemia or any previous history of arterial/venous thrombosis (including stroke, transient ischemic attack, myocardial infarction, pulmonary embolism, deep vein thrombosis, etc.); 16. Tendency of drug abuse or drug dependence, history of mental disorder or mental disorder with suicidal tendency; 17. Pregnant and lactating female; 18. Have participated in other clinical trials within 1 month; 19. The investigator judged that the patient's compliance was likely to be affected by the unstable condition of the disease, or the investigator judged that the patient should not participate in the study for other reasons.

Patients were consecutively enrolled based on identical inclusion and exclusion criteria.

***Sample Size***

The study was a single-arm design, and the primary efficacy endpoint was the overall response rate within 7 days of initiation treatment. According to the previous clinical trial data of the similar investigational drug, the overall response rate within 7 days after initiating treatment was about 70%, With a target overal response rate was no less than 50%, a statistical power of 80%, and a type I error (α) set at 0.025. The calculated sample size require was 47 cases, However, considering the safety assessment of the subjects, it is recommended to set the number of evaluable cases at 60, considering 20% dropout rate, and the total number of cases required was 72.

***Evaluation of efficacy***

***Primary Efficacy Endpoint***: Overall response (OR) proportion of participants within 7 days after starting treatment.

OR was defined as complete response (CR) or partial response (PR) after treatment.

OR proportion = (number of CR + number of PR) / total number of subjects × 100%.

***Secondary Efficacy Endpoints***

(1) The percentage of participants whose PLT reaches 50×10^9^/L and 100×10^9^/L or above within 7 days after starting treatment.

(2) The time (in days) it takes for PLT to first reach 30×10^9^/L and increase to more than twice the baseline level, 50×10^9^/L, and 100×10^9^/L within 7 days after starting treatment.

(3) The duration (in days) that the platelet count first reaches 30×10^9^/L and increases to more than twice the baseline level, 50×10^9^/L, and 100×10^9^/L within 7 days after starting treatment.

(4) The OR proportion (%) of participants on days 14, 21, and 28 after starting treatment.

(5) Bleeding scores before treatment and 7 days after treatment. Scoring according to the bleeding score system in the "Chinese Expert Consensus on the Diagnosis and Treatment of ITP in Adults (2016 Edition)".

**2. Description for medicine or protocol of treatment in detail of 5%IVIg Group**

Based on data from a national longitudinal cohort study on hematological diseases (NCT04645199), we identified 63 patients who received IVIg treatment during the same period, met the same inclusion and exclusion criteria as the 10% IVIg group, who received 5% IVIg therapy (0.4 g/kg/day for five consecutive days), and had both platelet monitoring data up to 28 days post-treatment and complete efficacy data. Baseline characteristics were compared between the two groups (Table 1), and no statistically significant differences were observed, supporting the validity of subsequent comparisons of efficacy and safety outcomes.

**3. The calculation of test power**

This single-arm, open-label, multicenter clinical trial of 10% IVIg for the treatment of ITP included a total of 69 patients. Based on the inclusion and exclusion criteria and treatment protocols of the 10% IVIg clinical trial, only 63 patients were concurrently enrolled in the 5% IVIg group from the national hematology disease longitudinal cohort study. To determine the comparability of the two data sets, we calculated the power. This study conducted a combined analysis using data results from independent clinical trial and cohort study, with an alpha (α) of 0.05. The sample size for the 10% IVIg group (N1) was 69, and for the 5% IVIg group (N2) was 63. The non-inferiority margin (D0) was set at -0.083, the actual difference observed (D1) was 0.045, and the proportion in the reference group (P2) was 0.825. Numeric results for the non-inferiority tests based on the difference (P1 - P2) were as follows: under the null hypothesis (H0) that P1 - P2 < D0, and the alternative hypothesis (H1) that P1 - P2 > D0. The Z-test (unpooled) calculated the power as 0.6693.

**Table S1 Bleeding scores of patients before and 7 days after treatment**

|  | Day 0 | | Day 7 | |
| --- | --- | --- | --- | --- |
|  | **5%IVIg**  (n=63) | **10%IVIg**  (n=69) | **5%IVIg**  (n=63) | **10%IVIg**  (n=67)^*^ |
| Bleeding score |  |  |  |  |
| 0 | 32（50.8） | 26 (37.7) | 58 (92.1) | 42 (62.7) |
| 1 | 23（36.5） | 31 (44.9) | 3 (4.8) | 16 (23.9) |
| 2 | 7（11.1） | 9 (13.0) | 2 (3.2) | 6 (9.0) |
| 3 | 1（1.6） | 3 (4.3) | 0 | 1 (1.5) |
| 4 | 0 | 0 | 0 | 1 (1.5) |
| 5 | 0 | 0 | 0 | 1 (1.5) |
| ≥6 | 0 | 0 | 0 | 0 |
| Median  (range), | 0 (0-3) | 0 (0-3) | 0 (0-2) | 0 (0-5) |
| *p**^[a]^ | ＜0.0001^[b]^ | 0.012^[c]^ | 0.131^[d]^ | <0.001^[e]^ |

* Two patients in the 10%IVIg group had no bleeding score on day 7.

[a] Using the rank sum test

[b] Comparison of bleeding scores before treatment and 7 days after treatment in the 5% IVIg group

[c] Comparison of bleeding scores before treatment and 7 days after treatment in the 10% IVIg group

[d] Comparison of bleeding scores on Day 0 between the 5% IVIg group and the 10% IVIg group

[e] Comparison of bleeding scores on Day 7 between the 5% IVIg group and the 10% IVIg group

**Table S2 Efficacy Rates After 7 Days of Treatment**

|  | **5%IVIg**  **(N = 63)** | **10%IVIg**  **(N = 69)** | ***p*** |
| --- | --- | --- | --- |
| OR proportion of Participants Within 7 Days After Starting Treatment, n/m (%) ^[a]^ | 52/63  (82.5) | 60/69  (87.0) | 0.643 |
| Results of Efficacy Evaluation of the Study Drug, n (%) ^[b]^ |  |  |  |
| CR | 32 (50.8) | 32 (46.4) | 0.873 |
| PR | 20 (31.7) | 28 (40.6) |  |
| NR | 11 (17.5) | 9 (13.0) |  |

[a] Using the Chi-squared test

[b] Using the Wilcoxon-Mann-Whitney Test

**Table S3 Duration of Platelet Count Maintenance After Treatment**

|  | **5%IVIg**  **(N = 63)** | | **10%IVIg**  **(N = 69)** | | ***p*** |  |
| --- | --- | --- | --- | --- | --- | --- |
| The duration of a first 30×10^9^/L platelet count that increased to more than twice the baseline level within 7 days after the initiation of treatment. Median days (IQR) | 19(12,26) | | 13(11,19) | | 0.044 |  |
| The duration of a first 50×10^9^/L platelet count within 7 days after the initiation of treatment. Median days (IQR) | 17(10,24) | | 13(6,19) | | 0.072 |  |
| The duration of a first 100×10^9^/L platelet count within 7 days after the initiation of treatment. Median days (IQR) | 10(9,24) | 11(10,17) | | 0.437 | | |

Using the rank sum test

**Table S4 Treatment-Emergent Adverse Event**

| AE, n (%) | 5%IVIg  （N=69）  n（%） | 10%IVIg  （N=72）  n（%） | *p*  （χ^2^ test） |
| --- | --- | --- | --- |
| Headache | 10（15.9） | 16（22.2） | 0.475 |
| White-cell count decreased | 7（11.1） | 17（23.6） | 0.122 |
| Neutrophil count decreased | 5（7.9） | 11（15.3） | 0.294 |
| Blood pressure increased | 5（7.9） | 8（11.1） | 0.740 |
| Hypokalemia | 4（6.3） | 7（9.7） | 0.690 |
| Dizziness | 4（6.3） | 6（8.3） | 0.913 |
| Anemia | 5（7.9） | 6（8.3） | 1.000 |
| Emesis | 3（4.8） | 5（6.9） | 0.865 |
| White blood cell count increased | 3（4.8） | 4（5.6） | 1.000 |
| Hypoalbuminaemia | 4（6.3） | 4（5.6） | 1.000 |
| Hypertriglyceridemia | 3（4.8） | 4（5.6） | 1.000 |
| Upper respiratory tract infection (URTI). | 2（3.2） | 4（5.6） | 0.802 |
| Rash | 2（3.2） | 4（5.6） | 0.802 |
| Fever | 4（6.3） | 4（5.6） | 1.000 |
| Insomnia | 3（4.8） | 4（5.6） | 1.000 |
